# Dairy Food Consumption is Inversely Associated with the Prevalence of Periodontal Disease in Korean Adults

**DOI:** 10.3390/nu11051035

**Published:** 2019-05-09

**Authors:** Kyueun Lee, Jihye Kim

**Affiliations:** Department of Medical Nutrition, Graduate School of East-West Medical Science, Kyung Hee University, Yongin 17104, Korea; kyueun07@khu.ac.kr

**Keywords:** periodontal disease, dairy foods, milk, yogurt

## Abstract

Dairy food consumption is known to be inversely associated with periodontal disease. However, there are conflicting results depending on the type of dairy foods. The aim of this study was to determine the relationship between individual dairy food consumption and periodontal disease. A total of 9798 Korean adults, aged ≥30 years, who participated in the fifth and sixth Korea National Health and Nutrition Examination Survey were included in this study’s analysis. Dairy food consumption was measured by the semi-quantitative food frequency questionnaire. Periodontal disease was defined as Community Periodontal Index score ≥3 in more than one of six sextants. Frequent intake of dairy foods (≥7 servings/week) was associated with a 24% lower prevalence of periodontal disease compared with never consumers after adjustment for age, gender, income, education, smoking, alcohol consumption, body mass index, diabetes mellitus status, calcium intake, tooth brushing frequency, and use of dental floss (Odds ratio (OR)= 0.76, 95% CI = 0.63–0.91, *p* for trend = 0.052). Also, frequent intake of milk (≥7 servings/week) was associated with a 26% lower prevalence of periodontal disease after adjustment for potential confounders (OR = 0.74, 95% CI = 0.61–0.89, *p* for trend = 0.022). Frequent consumption of dairy food including milk may have a beneficial effect on periodontal disease in the Korean adult population.

## 1. Introduction

Periodontal disease is characterized by microbial-associated, host-mediated inflammation, and it brings about the diminished density of alveolar bone as well as the destruction of bone structure [1]. Progression of periodontal disease is a major risk factor of tooth loss in adult populations. According to the Global Burden Disease Study 2015, the prevalence of periodontal disease increased by 25.4% from 2005 to 2015 [2] and the global cost was estimated at 442 billion USD. The data from the Korea National survey reported that the prevalence of periodontal disease was about 29.8% in Korean adults aged over 19 years and 47.2% for those aged over 50 years [3]. Thus, the burden of periodontal disease will likely be even more increased in a rapidly aging population. Diet and nutrition are associated with the risk of periodontal disease [4,5,6]. Recently, dairy products have been proposed to have a beneficial effect on periodontal disease due to the various nutrients and food components [7]. A few studies have examined the association between total dairy intake and periodontal disease, but the results were conflicting. Al-Zahrani et al., showed that an increased intake of dairy foods was inversely associated with the prevalence of periodontal disease [8]. However, Shimazaki et al. found that lactic acid foods, but not total dairy intake, was associated with periodontal disease [9]. Additionally, only one study has examined the relationship between different types of dairy foods and periodontal disease [10]; however, this study was conducted only in older adults aged ≥65 years, not the general population, and it had a very small sample size. 

Dairy foods are good sources of various nutrients including calcium, which may have beneficial effects on health outcome, such as bone development [11]. The content of calcium and other components (e.g., whey, casein, *Bifidobacterium*) may vary in different types of dairy products [12] and thus different types of dairy food products may have different effects on periodontal disease.

Therefore, this study was conducted to examine the associations between individual dairy consumption as well as total dairy intake and the risk of periodontal disease in the general Korean population using a large scale of nationally representative data examined recently.

## 2. Materials and Methods 

### 2.1. Study Population

**Figure 1:**
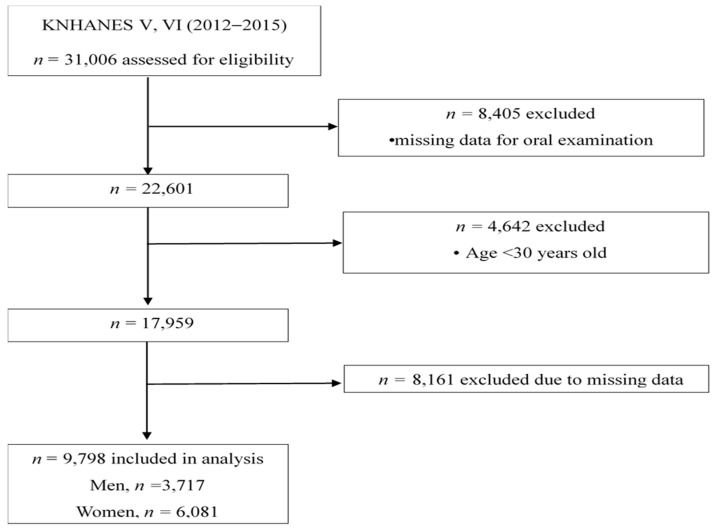
Flow chart for selection of study population.

This study was based on the fifth and sixth Korea National Health and Nutrition Examination Survey (KNHANES) conducted in 2012 and 2015 [13,14]. The KNHANES is a national survey, designed according to stratified, multi-stage, clustered probability sampling, which is conducted by the Korea Centers for Disease Control and Prevention. Among the 31,006 individuals (8058 in 2012, 8018 in 2013, 7550 in 2014, and 7380 in 2015) who participated in the survey, 17,959 adults aged between 30 and over 60 were included (Figure 1). Among them, 8161 subjects were further excluded because of insufficient data on dietary, socio-economic, and health-related behaviors. As a result, a total of 9798 Korean adults (3717 men and 6081 women) were included for the final analysis. The study was approved by the Institutional Review Board of Korea Centers for Disease Control and Prevention. Written informed consent was obtained from all participants.

### 2.2. Definition of Periodontal Disease

The oral examination of the fifth and sixth KNHANES were conducted by two members of the public health dentistry belonging to the Disease Control Headquarters and about 30 members of the public health dentistry supported by the city and province. Periodontal status was assessed by the Community Periodontal Index (CPI) of the World Health Organization [15]. The oral cavity was divided into six sites: upper right posterior, upper anterior, upper left posterior, lower right posterior, lower anterior, and lower left posterior. Probing depth was measured at six sites around each index tooth and the highest score was recorded. Six segments were evaluated for each mouth. The index teeth were numbers 17, 16, 11, 26, and 27 in the maxilla, and 47, 46, 31, 36, and 37 in the mandible. The CPI scores ranged from 0 to 4 and are defined as: 0, healthy; 1, bleeding on gentle probing; 2, dental calculus and bleeding; 3, shallow pockets of 4 or 5 mm; and 4, deep periodontal pockets of 6 mm or more [16]. Periodontal disease was defined as a CPI score ≥ 3 in more than one of six sextants [17] and severe periodontal disease was defined as CPI score ≥ 3 in all of six sextants.

### 2.3. Dietary Assessment

Dairy food consumption was measured using a validated semi-quantitative food frequency questionnaire [18] by trained dietitians through face-to-face interviews. Total dairy intake included milk and yogurt (liquid yogurt and curd yogurt). Cheese intake was not measured in the fifth and sixth KNHANES. Subjects were asked to answer about usual frequencies and serving sizes of milk intake (or yogurt) over the previous year. The questions were as follows: “How often have you consumed milk (or yogurt) during the past year?”. Answers were categorized into nine options for frequency: (1) never; (2) once a month; (3) 2–3 times a month; (4) once a week; (5) 2–4 times a week; (6) 5–6 times a week; (7) once a day; (8) twice a day; (9) 3 times a day and 3 options were provided for serving size: (1) 1/2 serving; (2) one serving; (3) one and a half servings. The frequency of milk and yogurt consumption was multiplied by serving size. Consequently, dairy consumption was categorized into five groups as follows: (1) never; (2) ≤1; (3) 1<–≤3; (4) 3<–<7; (5) ≥7 servings/week. One serving of dairy foods was regarded as 200 ml for milk and 100 ml for yogurt [19]. Calcium intake was evaluated by the 24-h recall method and was estimated using the food composition table of the Rural Development Administration [20].

### 2.4. Covariates

Information on socio-demographic characteristics and health-related behaviors was obtained by trained staffs using in-person interviews. Education level was categorized into three groups: ≤6 years (elementary school level), 7–12 years (middle or high school level), and >12 years (college graduate or more). Income was divided into quartiles based on age and sex. The second and third quartiles were combined in order to represent the middle level. Smoking status was divided into three groups: non-smokers, former smokers, and current smokers. Alcohol consumption was divided into never-drinker, moderate drinker (<2 times/week), and heavy drinker (≥2 times/week). Body mass index (BMI) was calculated as weight (kg) divided by height squared (m^2^). Diabetes mellitus status was defined as fasting blood glucose ≥ 126 mg/dL or use of oral hypoglycemic medication, or insulin injection. In terms of oral health behaviors, the frequency of tooth brushing and the use of dental floss were examined. The frequency of tooth brushing was measured as follows: before and after breakfast, before and after lunch, before and after dinner, after snack, and before sleep. The frequency of tooth brushing was categorized into two groups (<3 times a day, ≥3 times a day). 

### 2.5. Statistical Analysis

All data was analyzed using SAS version 9.4 (SAS institute, Cary, NC, USA). The number of sample units was considered to calculate the pooled weight variable. Data are presented as the numbers and percentages (categorical) or as means ± standard errors (continuous). Differences in general characteristics between periodontal disease and normal groups were examined with chi-square tests for categorical variables or Student’s *t*-tests for continuous variables. Differences in variables according to the dairy food consumption were tested using the chi-square test for categorical variables, or the PROC SURVEYREG procedure for continuous variables [21], as appropriate. Multivariate logistic regression analysis was performed to determine the odds ratios (ORs) and 95% confidence intervals (CIs) for the risk of periodontal disease according to total dairy consumption, milk consumption, or yogurt consumption. Model 1 was unadjusted. Model 2 was adjusted for age and sex, and model 3 was adjusted for age, sex, education level, income level, smoking status, alcohol consumption, calcium intake, frequency of tooth brushing, and use of dental floss. In this study, *p* values < 0.05 were considered statistically significant. 

## 3. Results

### 3.1. Characteristics of Subjects

General characteristics of subjects according to the presence of periodontal disease are shown in Table 1. About 28.3% of participants were defined as having periodontal disease. Participants with periodontal disease were older, more likely to be men, less educated, had lower income, had higher BMI, had higher prevalence of diabetes mellitus, were more likely to be current smokers and heavy alcohol drinkers, had less tooth brushing, were less likely to use dental floss, and had lower intakes of dairy foods than normal subjects (all *p* < 0.001). However, calcium intake did not differ between the normal group and periodontal group.

### 3.2. Characteristics of Subjects According to Dairy Food Consumption

Characteristics of subjects according to total dairy consumption and milk consumption are shown in Table 2 and Table 3, respectively. Subjects in the highest category of total dairy intake and milk intake had lower prevalence of periodontal disease, were younger, were more likely to be women, were more educated, had higher income level, were more likely to be non-smokers, were less likely to be moderate alcohol drinkers, had lower prevalence of diabetes mellitus, had more tooth brushing (≥3times/day), and were more likely to use dental floss compared with those in the lowest category (all *p* < 0.001). Subjects in the highest category of total dairy intake and milk intake had higher intake of calcium than those in the lowest consumption (*p* < 0.001). 

### 3.3. Association of Dairy Food Consumption with Periodontal Disease

The odds ratios (ORs) and 95% confidence intervals (CIs) for risk of periodontal disease according to total dairy intake, milk intake, and yogurt intake are shown in Table 4. The unadjusted OR for periodontal disease decreased by 52% in frequent consumers of dairy (≥7 servings/week) compared with non-consumers (OR = 0.48, 95% CI = 0.41–0.57, *p* for trend < 0.001) (Model 1). This trend remained even after adjustment for age, sex, income, education, smoking, alcohol intake, calcium intake, brushing frequency, and use of floss (OR = 0.76, 95% CI: 0.63–0.91, *p* for trend = 0.052) (Model 3).

The unadjusted OR for periodontal disease decreased by 48% in frequent consumers of milk (≥ 7 servings/week) compared with non-consumers (OR = 0.52, 95% CI = 0.44–0.62, *p* for trend < 0.001) (Model 1). This trend maintained even after adjustment for potential confounders such as age, sex, income, education, smoking, alcohol, BMI, diabetes mellitus status, calcium, brushing frequency, and use of dental floss (OR = 0.74, 95% CI: 0.61–0.89, *p* for trend = 0.022) (Model 3). In terms of yogurt, the unadjusted OR for periodontal disease decreased according to the increase in yogurt consumption (OR = 0.65, 95% CI: 0.53–0.80, *p* for trend < 0.001) (Model 1). However, this trend disappeared after adjustment for potential confounding factors (OR = 0.83, 95% CI: 0.67–1.05, *p* for trend = 0.188) (Model 3). 

## 4. Discussion

We found that dairy food consumption was inversely associated with the prevalence of periodontal disease in Korean adults. Higher total dairy intake and milk intake were associated with 25% lower risks of periodontal disease, after adjusting for potential confounders, such as age, sex, education, income level, smoking status, alcohol consumption, BMI, diabetes mellitus status, calcium intake, tooth brushing frequency, and use of dental floss. These results suggest that frequent intake of dairy foods including milk may have a protective effect on periodontal disease.

Our results are in line with previous findings. Adegboye et al., reported that frequent consumption of both dairy foods and milk was associated with a 4% lower risk of periodontitis, as defined as the number of teeth with attachment loss ≥ 3 mm in older Danish adults (aged over 65 years) [10]. The data from the National Health and Nutrition Examination Survey found that individuals in the highest quintile of dairy intake had a 20% lower prevalence of periodontal disease (defined as probing pocket depth ≥ 4 mm and clinical attachment loss ≥ 3 mm [22]) than those in the lowest quintile in US adults over 18 years after adjusting for age, gender, ethnicity, smoking, education, diabetes, poverty index, vitamin use, body mass index, and physical activity [8]. 

The mechanism behind the inverse association between dairy food intake and periodontal disease has not been revealed yet. Dairy foods are good sources of various nutrients needed for bone development and maintenance, such as calcium, magnesium, phosphorus, potassium, vitamin D, and proteins. For example, dairy calcium may have a favorable effect on periodontal health by enhancing alveolar bone density [23]. High calcium intake from dairy foods may prevent bone loss by inhibiting the secretion of parathyroid hormone, which contributes to bone resorption [24]. A clinical trial showed that calcium supplementation of 500 mg/day for 3 months significantly increased alveolar bone density and reduced gingival inflammation in Indian adults aged 35–55 years [25]. Dairy proteins, such as whey protein and lactoferrin, may help with the prevention of periodontal disease. Whey protein may prevent alveolar bone loss by increasing hydroxyproline, which can strengthen the coherence of bone [26]. A rat study proved that alveolar bone loss was significantly decreased in the 1.0% whey protein powder group compared to the control group [27]. Additionally, lactoferrin, an iron-binding glycoprotein in milk, may have a favorable effect on periodontal disease. In an intervention study, *Porphyromonas gingivalis* and *Fusobacterium nucleatum*, the causative bacteria of periodontal disease and tooth decay, were significantly decreased in a treatment group with lactoferrin compared to the control group [28]. 

Fermented dairy products, such as yogurt, contain lactic acid. Lactic acid may help the inhibition of bacteria growth or result in the interference of the activities of periodontal pathogens by decreasing oral pH [29]. The probiotic bacteria from lactic acid, such as *Lactobacillus* and *Bifidobacterium*, may have a protective effect on periodontal disease by inhibiting excessive growth of periodontal pathogens and by stimulating the immune system [30]. Shimazaki et al. reported that individuals consuming lactic acid foods including yogurt and lactic acid drinks (≥55 g/day) had lower values in both mean probing depth and severe clinical attachment loss than those not eating these foods after adjusting for potential risk factors in Japanese adults aged 40 to 79 years [9]. These beneficial components of dairy foods may have synergistic favorable effects on periodontal disease.

In the present study, yogurt consumption tended to be associated with lower prevalence of periodontal disease, even though statistical significance was not reached. The different result may be due to lower intake of yogurt in the Korean population. In European countries, the majority of people consumed more than 1 serving (100 g) of yogurt per day and more than one-third of the population had 5 servings of yogurt [31], whereas Koreans, on average, consumed only 13 grams of yogurt per day [32]. 

The association between cheese consumption and periodontal disease was not examined in this study because cheese intake was not measured in KNHANES. Previously, two cross-sectional studies examined the association in Danish adults aged 65 years and older and Japanese adults aged 40 to 75 years [9,10]. Neither of the studies showed a significant association between cheese intake and periodontal disease.

Several limitations exist in this study. First, the results cannot prove a causal relationship between dairy intake and periodontal disease because the study was designed cross-sectionally. Second, there is a difficulty in applying results to other age groups, since children and adolescents are not included in the analysis. Third, the prevalence of periodontal disease could be different depending on the criteria used to diagnose periodontal disease. Various criteria for diagnosis of periodontal disease, such as probing pocket depth and clinical attachment loss, can be used, but periodontal disease was diagnosed based on only probing pocket depth in this study. Lastly, oral hygiene index (e.g., Simplified Oral Hygiene Index and Plaque Index) and the number of present teeth, which might have affected periodontal disease, were not measured in this study. Despite these limitations, this study was the first study to explore the effect of individual dairy foods such as milk or yogurt as well as overall dairy foods on periodontal disease. In addition, dietary assessment was evaluated by semi-quantitative food frequency questionnaire and thus the habitual dairy intake of subjects could be compared with other studies. Also, this study included specific potential confounders for oral health behaviors such as tooth brushing frequency and the use of dental floss in the analysis. 

## 5. Conclusions

This study showed that frequent consumption of dairy foods and milk was associated with lower prevalence of periodontal disease after adjustment for potential confounders among the general Korean population. Therefore, the result suggests that dairy food consumption including milk should be recommended in order to prevent periodontal disease. Further research should be performed to reveal the mechanism behind the effect of dairy foods on periodontal disease. Also, randomized clinical trials should be done to confirm the effect of dairy foods by type and their components on periodontal disease.

## Figures and Tables

**Table 1:** General characteristics of participants ^1^.

Characteristics	Periodontal Disease (*n* = 2771)	Normal (*n* = 7027)	*p* Value
Age (years)	49.4 ± 0.2	44.3 ± 0.2	<0.001
Sex			<0.001
Men	1397 (60.9)	2320 (44.3)	
Women	1374 (39.1)	4707 (55.7)	
Education level (%)			<0.001
≤6 years	549 (15.2)	703 (7.6)	
7–12 years	1444 (53.7)	3188 (45.3)	
>12 years	778 (31.1)	3136 (47.1)	
Income level (%)			<0.001
Low	749 (27.3)	1517 (22.3)	
Middle	1395 (50.4)	3580 (50.8)	
High	627 (22.3)	1930 (26.8)	
Smoking status (%)			<0.001
Non-smokers	1397 (41.9)	4798 (60.1)	
Former smokers	606 (24.0)	1162 (19.6)	
Current smokers	768 (34.1)	1067 (20.2)	
Alcohol consumption (%)			<0.001
Never	725 (22.2)	1777 (22.0)	
<2 times/week	1333 (48.2)	3940 (55.8)	
≥2 times/week	713 (29.7)	1310 (22.3)	
Body mass index (kg/m^2^)	24.6 ± 0.1	23.8 ± 0.1	<0.001
Diabetes mellitus (%)	409 (14.3)	425 (5.5)	<0.001
Tooth brushing frequency (%)			
<3 times/day	1445 (52.1)	2903 (42.0)	
≥3 times/day	1326 (47.9)	4124 (58.0)	
Use of dental floss (%)	436 (9.8)	1151 (17.9)	<0.001
Total dairy intake ^2^ (servings/week)	3.7 ± 0.1	4.7 ± 0.1	<0.001
Milk intake (servings/week) ^2^	2.0 ± 0.1	2.7 ± 0.1	<0.001
Yogurt intake (servings/week)	1.7 ± 0.1	2.0 ± 0.1	<0.001
Calcium intake (mg/day)	532.2 ± 7.4	528.7 ± 5.2	0.683

^1^ All values are means ± standard errors or numbers (percentages). Comparisons of variables between two groups were tested with chi-square test for categorical variables or Student-*t* test for continuous variables. ^2^ One serving was equal to 200 mL of milk and 100 mL of yogurt.

**Table 2:** General characteristics of participants according to total dairy food consumption ^1^.

Total Dairy Food Consumption (Servings/Week)						
Characteristics	Never	0–≤1	1<–≤3	3<–<7	≥7	*p* Value
No. of subjects	1439	1799	1930	2058	2572	
Normal (%)	901 (61.4)	1238 (68.4)	1404 (72.8)	1522 (73.7)	1962 (76.7)	<0.001
Mild periodontal disease (%)	522 (37.5)	547 (30.6)	513 (26.4)	523 (25.5)	596 (22.8)	
Severe periodontal disease (%)	16 (1.1)	14 (1.0)	13 (0.9)	13 (0.8)	14 (0.5)	
Age (years)	48.9 ± 0.3	46.6 ± 0.3	45.0 ± 0.2	44.5 ± 0.3	45.0 ± 0.2	<0.001
Sex (%)						<0.001
Men	654 (55.4)	736 (51.7)	765 (51.5)	762 (48.4)	800 (41.9)	
Women	785 (44.6)	1063 (48.3)	1165 (48.5)	1296 (51.6)	1772 (58.1)	
Education level (%)						<0.001
≤6 years	345 (18.6)	284 (12.3)	201 (8.3)	186 (6.5)	236 (6.8)	
7–12 years	703 (51.8)	905 (50.8)	880 (44.5)	934 (45.7)	1210 (47.2)	
>12 years	391 (29.6)	610 (36.9)	849 (47.2)	938 (47.9)	1126 (46)	
Income level (%)						<0.001
Low	417 (30.2)	487 (27.9)	439 (22.7)	428 (21.2)	495 (20)	
Middle	718 (49.6)	918 (50.8)	1005 (51.6)	1092 (53)	1242 (48.7)	
High	304 (20.3)	394 (21.3)	486 (25.7)	538 (25.8)	835 (31.3)	
Smoking status (%)						<0.001
Never	783 (45.8)	1090 (51.9)	1201 (54.1)	1341 (57)	1780 (61.2)	
Former	310 (24)	357 (22.7)	348 (20.9)	339 (18.9)	414 (19.4)	
Current	346 (30.2)	352 (25.3)	381 (25)	378 (24.1)	378 (19.3)	
Alcohol consumption (%)						<0.001
Never	401 (23.5)	466 (22.3)	444 (19.6)	524 (22.6)	667 (22.4)	
<2 times/week	642 (45.4)	931 (52)	1081 (55.4)	1122 (53.5)	1497 (58)	
≥2 times/week	396 (31.2)	402 (25.7)	405 (24.9)	412 (23.9)	408 (19.6)	
Body mass index (kg/m^2^)	24.3 ± 0.1	24.1 ± 0.1	23.9 ± 0.1	24.0 ± 0.1	23.8 ± 0.1	0.016
Diabetes mellitus (%)	188 (12.2)	172 (8.6)	160 (8.1)	132 (6.1)	182 (6.7)	<0.001
Tooth brushing frequency (%)						<0.001
<3 times/day	769 (51.6)	897 (50.8)	855 (45.4)	881 (44.2)	946 (36.8)	
≥3 times/day	670 (48.4)	902 (49.2)	1075 (54.6)	1177 (55.8)	1626 (63.2)	
Use of dental floss (%)	227 (15.8)	421 (23.3)	541 (26.8)	611 (29.1)	771 (30.6)	<0.001
Calcium intake (mg/day)	460.8 ± 9.5	467.0 ± 9.7	498.2 ± 7.7	538.5 ± 7.7	632.2 ± 9.8	<0.001

^1^ All values are means ± standard errors or numbers (percentages). Comparisons of the variables across dairy consumption were made using either chi-square tests (categorical variables) or PROC SURVEYREG procedure (continuous variables).

**Table 3:** General characteristics of participants according to milk consumption ^1^.

Milk Consumption (Servings/Week)						
Characteristics	Never	≤1	1<–≤3	3<–< 7	≥7	*p* Value
No. of subjects	2949	2623	1885	793	1548	
Normal (%)	1953 (65.3)	1863 (70.8)	1421 (74.7)	595 (75.5)	1195 (78.4)	0.001
Mild periodontal disease (%)	968 (33.5)	743 (28.4)	459 (25.0)	189 (25.0)	342 (22.8)	
Severe periodontal disease (%)	28 (1.2)	17 (0.8)	5 (0.3)	9 (1.3)	11 (0.7)	
Age (years)	48.4 ± 0.2	45.6 ± 0.2	44.7 ± 0.3	43.1 ± 0.4	43.8 ± 0.3	<0.001
Sex (%)						<0.001
Men	1194 (50.5)	1070 (52.5)	643 (45.5)	348 (55.5)	462 (40.6)	
Women	1755 (49.5)	1553 (47.5)	1242 (54.5)	445 (44.5)	1086 (59.4)	
Education level (%)						<0.001
≤6 years	592 (15.5)	292 (8.8)	159 (6.6)	66 (5.9)	143 (6.8)	
7–12 years	1446 (51)	1283 (48.2)	857 (45.4)	359 (45)	687 (44.6)	
>12 years	911 (33.6)	1048 (43)	869 (48)	368 (49.1)	718 (48.6)	
Income level (%)						<0.001
Low	790 (27.5)	622 (24.4)	382 (20.2)	167 (22.2)	305 (20.6)	
Middle	1438 (48.5)	1359 (51.5)	1006 (53.8)	419 (52.3)	753 (48.9)	
High	721 (24)	642 (24.1)	497 (26)	207 (25.6)	490 (30.4)	
Smoking status (%)						<0.001
Never	1789 (52.3)	1600 (52)	1281 (60.6)	475 (52)	1050 (59.6)	
Former	574 (21.9)	494 (22)	302 (19.4)	147 (19.9)	251 (19.5)	
Current	586 (25.8)	529 (26)	302 (20)	171 (28.1)	247 (20.9)	
Alcohol consumption (%)						<0.001
Never	850 (24.7)	630 (20.5)	428 (20.1)	194 (21.1)	400 (22.5)	
<2 times/week	1403 (48.1)	1436 (54.1)	1095 (56.9)	432 (54.7)	907 (58.4)	
≥2 times/week	696 (27.2)	557 (25.4)	362 (23)	167 (24.2)	241 (19.1)	
Body mass index (kg/m^2^)	24.0 ± 0.1	24.0 ± 0.1	24.1 ± 0.1	24.0 ± 0.1	23.8 ± 0.1	0.501
Diabetes mellitus (%)	305 (9.6)	224 (8.5)	138 (7.1)	50 (4.9)	117 (6.8)	0.001
Tooth brushing frequency (%)						<0.001
<3 times/day	1454 (49)	1243 (47.8)	754 (41.6)	332 (43)	565 (36.9)	
≥3 times/day	1495 (51)	1380 (52.2)	1131 (58.4)	461 (57)	983 (63.1)	
Use of dental floss (%)	628 (21.8)	661 (23.7)	598 (31.3)	220 (28)	464 (30.2)	<0.001
Calcium intake (mg/day)	474.0 ± 7.1	487.6 ± 7.4	536.1 ± 8.2	600.1 ± 12.9	661.7 ± 12.2	<0.001

^1^ All values are means ± standard errors or numbers (percentages). Comparisons of the variables across milk consumption were made using either chi-square tests (categorical variables) or PROC SURVEYREG procedure (continuous variables).

**Table 4:** Odds ratios (ORs) and 95% confidence intervals (CIs) for the risk of periodontal disease according to dairy food consumption ^1^.

Dairy Food Consumption (Servings/Week)						
**Total dairy**	**Never**	**≤1**	**1<–≤3**	**3<–<7**	**≥7**	***p* for trend**
No. of subjects (No. of cases)	1439 (538)	1799 (561)	1930 (526)	2058 (536)	2572 (610)	
Model 1	1.00	0.73 (0.62–0.87)	0.59 (0.50–0.70)	0.57 (0.48–0.67)	0.48 (0.41–0.57)	<0.001
Model 2	1.00	0.85 (0.70–1.02)	0.75 (0.63–0.89)	0.75 (0.63–0.90)	0.64 (0.54–0.77)	<0.001
Model 3	1.00	0.91 (0.76–1.10)	0.84 (0.70–1.01)	0.87 (0.73–1.04)	0.76 (0.63–0.91)	0.052
**Milk**	**Never**	**≤1**	**1<–≤3**	**3<–<7**	**≥7**	***p* for trend**
No. of subjects (No. of cases)	2949 (996)	2623 (760)	1885 (464)	793 (198)	1548 (353)	
Model 1	1.00	0.78 (0.68–0.88)	0.64 (0.55–0.74)	0.61 (0.50–0.74)	0.52 (0.44–0.62)	<0.001
Model 2	1.00	0.89 (0.77–1.02)	0.80 (0.69–0.94)	0.78 (0.64–0.96)	0.71 (0.59–0.85)	<0.001
Model 3	1.00	0.90 (0.78–1.04)	0.88 (0.75–1.03)	0.82 (0.67–1.02)	0.74 (0.61–0.89)	0.022
**Yogurt**	**Never**	**≤1**	**1<–≤3**	**3<–<7**	**≥7**	***p* for trend**
No. of subjects (No. of cases)	3307 (1099)	2617 (693)	1871 (483)	1155 (285)	848 (211)	
Model 1	1.00	0.70 (0.61–0.81)	0.65 (0.56–0.76)	0.64 (0.53–0.77)	0.65 (0.53–0.80)	<0.001
Model 2	1.00	0.78 (0.68–0.91)	0.80 (0.69–0.93)	0.72 (0.59–0.88)	0.68 (0.55–0.84)	<0.001
Model 3	1.00	0.87 (0.74–1.01)	0.92 (0.79–1.08)	0.83 (0.67–1.01)	0.83 (0.67–1.05)	0.188

^1^ Adjusted ORs and 95% CIs estimated using a multiple survey logistic regression model. Model 1 was unadjusted. Model 2 was adjusted for age and sex. Model 3 was adjusted for covariates in model 2 plus further adjusted for income, education, alcohol consumption, BMI, diabetes mellitus status, calcium, tooth brushing frequency, and use of dental floss.
